# Green spaces and mortality due to cardiovascular diseases in the city of Rio de Janeiro

**DOI:** 10.11606/S1518-8787.2018052000290

**Published:** 2018-04-20

**Authors:** Ismael Henrique da Silveira, Washington Leite Junger

**Affiliations:** I Universidade do Estado do Rio de Janeiro. Instituto de Medicina Social. Programa de Pós-Graduação em Saúde Coletiva. Rio de Janeiro, RJ, Brasil; II Universidade do Estado do Rio de Janeiro. Instituto de Medicina Social. Departamento de Epidemiologia. Rio de Janeiro, RJ, Brasil

**Keywords:** Cardiovascular Diseases, mortality, Green Areas, Urban Health, Ecological Studies, Doenças Cardiovasculares, mortalidade, Áreas Verdes, Saúde da População Urbana, Estudos Ecológicos

## Abstract

**OBJECTIVE:**

Investigate the association between exposure to green spaces and mortality from ischemic heart and cerebrovascular diseases, and the role of socioeconomic status in this relationship, in the city of Rio de Janeiro, Brazil.

**METHODS:**

Ecological study, with the census tracts as unit of analysis. This study used data from deaths due to ischemic heart and cerebrovascular diseases among residents aged over 30 years, from 2010 to 2012. Exposure to green was estimated using the Normalized Difference Vegetation Index based on satellite images. The associations between exposure to green spaces and mortality rates due to ischemic heart and cerebrovascular diseases, standardized by gender and age, were analyzed using conditional autoregressive models, adjusted for the density of light and heavy traffic routes, pollution proxy, and by the socioeconomic situation, measured by the Social Development Index. Analyzes stratified by socioeconomic levels were also carried out, given by the tertiles of the Social Development Index.

**RESULTS:**

Among the greener sectors, with a Normalized Difference Vegetation Index above the third quartile, the reduction in mortality due to ischemic heart disease was 6.7% (95%CI 3.5–9.8) and cerebrovascular was 4.7% (95%CI 1.2–8.0). In the stratified analysis, the protective effect of green spaces on ischemic heart disease mortality was observed among the greenest sectors of all strata, and it was higher for those with a lower socioeconomic level (8.6%, 95%CI 1.8–15.0). In the case of mortality due to cerebrovascular diseases, the protective effect was verified only for the greenest sectors of the lowest socioeconomic level (9.6%, 95%CI 2.3–16.5).

**CONCLUSIONS:**

Mortality rates for ischemic heart and cerebrovascular diseases are inversely associated with exposure to green spaces when controlling socioeconomic status and air pollution. The protective effect of green spaces is greater among the tracts of lower socioeconomic level.

## RESUMO

**OBJETIVO:**

Investigar a associação entre a exposição aos espaços verdes e a mortalidade por doenças isquêmicas do coração e cerebrovasculares, e o papel do nível socioeconômico nessa relação, no município do Rio de Janeiro, Brasil.

**MÉTODOS:**

Estudo ecológico, tendo os setores censitários como unidade de análise. Foram utilizados os dados de óbitos por doenças isquêmicas do coração e cerebrovasculares, entre residentes com idade acima de 30 anos, ocorridos de 2010 a 2012. A exposição ao verde foi estimada por meio do Índice de Vegetação por Diferença Normalizada, baseado em imagens de satélite. As associações entre a exposição aos espaços verdes e a taxas de mortalidade por doenças isquêmicas do coração e cerebrovasculares, padronizadas por sexo e idade, foram analisadas por meio de modelos condicionais autorregressivos, ajustados pelas densidades de vias de tráfego leve e pesado, *proxy* de poluição, e pela situação socioeconômica, mensurada pelo Índice de Desenvolvimento Social. Também foram realizadas análises estratificadas por níveis socioeconômicos, dados pelos tercis do Índice de Desenvolvimento Social.

**RESULTADOS:**

Entre os setores mais verdes, com Índice de Vegetação por Diferença Normalizada acima do terceiro quartil, a redução da mortalidade por doenças isquêmicas do coração foi de 6,7% (IC95% 3,5–9,8) e por cerebrovascular foi de 4,7% (IC95% 1,2–8,0). Na análise estratificada, o efeito protetor dos espaços verdes na mortalidade por doenças isquêmicas do coração foi observado entre os setores mais verdes de todos os estratos, sendo maior para os de menor nível socioeconômico (8,6%; IC95% 1,8–15,0). No caso da mortalidade por doenças cerebrovasculares, o efeito protetor foi verificado apenas para os setores mais verdes do nível socioeconômico mais baixo (9,6%; IC95% 2,3–16,5).

**CONCLUSÕES:**

As taxas de mortalidade por doenças isquêmicas do coração e cerebrovasculares são inversamente associadas à exposição aos espaços verdes, controlando o nível socioeconômico e a poluição do ar. O efeito protetor dos espaços verdes é maior entre os setores de nível socioeconômico mais baixo.

## INTRODUCTION

Cardiovascular diseases (CVD) are among the major global health problems. According to the World Health Organization (WHO), of the 56 million deaths worldwide in 2012, more than 17.5 million (31%) were attributed to CVD, of which 7.4 million (13%) were caused by ischemic heart disease (IHD) and 6.7 million (12%) by cerebrovascular diseases (CBVD)^1^. In Brazil, in 2013, CVD was the cause of 339,672 deaths, 28% of the total. It was the second leading cause of death among people aged 20–59, behind only external causes, and the number one cause among people over 60^a^.

Traditionally, epidemiological studies on CVD have focused on behavioral and biological risk factors related to the individual^2^. Its high burden has been attributed mainly to the high prevalence of modifiable factors such as smoking, harmful alcohol use, inadequate diet and physical inactivity, precursors of hypertension, hyperlipidemia, and diabetes^1^ ^,^ ^3^ ^,^ ^4^. However, there has been an increasing interest in epidemiological studies on the influence of contextual determinants related to residential environment^2^ ^,^ ^5^.

Among the contextual or environmental factors that influence health, exposure to green spaces has been widely researched^6^. Green spaces refer to spaces with vegetation, such as forests, parks, squares, gardens, and tree-lined streets. Several studies in environmental epidemiology have observed the benefits of exposure to green spaces for mental health, increased practice of physical activities and reduction of general morbidity and mortality, and for specific causes, such as CVD^6^ ^–^ ^8^.

Despite evidence of the benefits of green spaces for health, studies on the subject in low- and middle-income countries are rare, especially in the Latin America region, whose proportion of the urban population already reaches 80% and tends to continue to increase^9^. Given this scenario, investigations into the influence of environmental factors on the health of the population are important to guide health promotion measures in the urban environment. The objective of this study was to investigate the association between exposure to green spaces and mortality from IHD and CBVD, and the role of socioeconomic level in this relationship, in the city of Rio de Janeiro, Brazil.

## METHODS

This ecological study was carried out in the city of Rio de Janeiro between 2010 and 2012, with the census tracts as units of analysis. In 2010, the population of the city was 6,320,446 inhabitants, the Municipal Human Development Index was 0.799, *per capita* income was R$1,492.63 and the Gini Index was 0.62, reflecting a high inequality in income distribution^b^.

The territory of the city consists of 10,504 census tracts, of which 7,922 are classified as normal, 2,212 are classified as subnormal agglomerates, corresponding to areas of irregular settlements, and the rest comprises sectors with no population or classified as hospitals, orphanages, prisons, etc. The analyzes considered only the normal sectors due to the higher occurrence of losses, during georeferencing, in subnormal agglomerates.

Mortality data were obtained from the Mortality Information System (SIM) of the Municipal Health Department of Rio de Janeiro. The deaths of persons aged over 30 years old and whose basic causes were IHD and DCBV, grouped in Chapter IX of the 10th Review of the International Classification of Diseases (ICD-10), respectively, between codes I20 to I25 and I60 to I69.

Death addresses were georeferenced in three stages: address standardization, georeferencing, and manual intervention. The first two stages were programmed in R. In standardization, the most frequent errors and abbreviations have been replaced. In the georeferencing stage, the geographical coordinates of the addresses were obtained from the Google Maps address database, through the Google Application Programming Interface (API), a set of preprogrammed functions such as georeferencing. Finally, the manual intervention was performed to locate the addresses not found initially.

After georeferenced, deaths were aggregated by census tract. The cartographic base of census tracts, relative to the 2010 census, was obtained from the Brazilian Institute of Geography and Statistics (IBGE). Standardized mortality ratios (SMR) for IHD and CBVD, by gender and age group, were calculated by dividing the number of deaths observed by the number of deaths expected in each unit of analysis. The expected values were calculated based on the mortality coefficients of the city, by the respective causes and period, by gender and age group.

Exposure to green spaces was measured using the Normalized Difference Vegetation Index (NDVI). The NDVI is made up of satellite images, serving to characterize the vegetation density of a region. The chlorophyll pigments present in the plants produce a low red reflectance (RED) and high reflectance near infrared (NIR) pattern. The index is determined by the equation:

NDVI=(NIR−RED)/(NIR+RED)

The NDVI can range from -1 to 1. Negative values indicate the presence of ice, water, and clouds; values around -0.1 and 0.1 correspond to the surfaces discovered; and values close to 0.7 refer to dense vegetation^c^.

The images were obtained from the Landsat 4-5TM collection of the United States Geological Survey (USGS), with a resolution of 30 meters. Only images with less than 10% of clouds generated in the period were used, totaling seven. First, the average NDVI of the period was calculated and, then, the average NDVI of each census sector was calculated considering buffers of 100 meters from its edges. The buffers were used so we could consider the exposure around the sector and not just closed to its limit.

Densities of vehicle traffic roads were used as indirect measures of air pollution, according to other epidemiological studies^10^ ^,^ ^11^. The roads were separated into two groups of traffic: light and heavy. Light traffic roads are those classified as local and collectors, responsible for the distribution of internal traffic to neighborhoods. Heavy traffic roads, classified as structural, primary arterial and secondary arterial, establish fast connections, serve distant displacements and are used by a large volume of vehicles. The density was obtained by the quotient between the length of the roads and the area that includes the census section and a buffer of 100 meters from its edges. The digital cartographic base of the sites was obtained from the Instituto Pereira Passos of the City Hall of Rio de Janeiro.

The socioeconomic level was measured through the Social Development Index (SDI), which expresses the degree of development of a given area^12^. It consists of eight indicators, based on data from the 2010 Census, namely: proportion of households with water supply through the general network, proportion of households with sewage collection via the general or pluvial network, proportion of households with garbage collection, average number of toilets per person, proportion of illiteracy among persons aged 10 to 14 years, average income of persons responsible for the household (minimum wages), proportion of persons responsible for the household with income of up to two minimum wages and proportion of persons responsible for the domicile with income equal to or greater than 10 minimum wages.

To calculate the index, the indicators were first normalized, so that they became compatible, varying between zero and one. Subsequently, the arithmetic mean of these indicators was calculated, which corresponds to the SDI value.

A binary variable was created to indicate the census tracts located on the coast. These tracts are generally areas with better socioeconomic conditions and presence of vegetation; but due to the sand strip, the value of average NDVI is reduced.

The associations between green spaces and SMR due to IHD and CBVD were analyzed using conditional autoregressive (CAR) models with a Gaussian distribution. The outcome variables analyzed in the models were the log of SMR by IHD and CBVD to approach a Gaussian distribution. The models were adjusted by SDI, the density of light traffic roads, the density of heavy traffic roads, coastal tract indicator and average of NDVI (categorized in quartiles). The relative risk value can be obtained by the exponential of the regression coefficients.

The analyzes were carried out in two stages: considering all sectors included in the study and stratified by SDI tertiles (to investigate the effect modification of the socioeconomic level). The strata were classified as: low (SDI from 0.264 to 0.586); intermediate (SDI from 0.586 to 0.642); and high (SDI from 0.642 to 0.900). Spatial autocorrelation was evaluated using the Moran I Index of multivariate linear regression residuals. The models with spatial dependence were maintained in all the analyzes to avoid the underestimation of the variance of the regression coefficient estimators.

The CAR model is used in situations where spatial autocorrelation is present. It is specified for a set of conditional probability distributions of each observation, considering the values of the others. Assuming that {Y(si): si ∈ (s1… sn)} is a Gaussian process in which {s1… sn} forms the set of areas S, the model can be defined by specifying the mean and variance, according to equations^13^:

$$\begin{array}{l} \text{E}[\text{Y}({\text{s}}_{i})|\text{Y}({\text{s}}_{\left(-\text{i}\right)})]=x(s_{i})’\text{β}+\sum_{\text{j}=\text{i}}^{\text{n}}{\text{c}}_{\text{ij}}[\text{Y}({\text{s}}_{j})-\text{x}({\text{s}}_{i})’\text{β},\\ \text{Var}[Y(s_{\text{i}})|\text{Y}({\text{s}}_{\left(-\text{i}\right)})]={{\text{σ}}_{i}}^{2},\text{sendo}i=1,\;\ldots ,\text{N}, \end{array}$$

Where $\text{Y}({\text{s}}_{(-\text{i)}})=\{\text{Y}({\text{s}}_{\text{j}}):\text{j}\neq \text{i}\},\text{Y}({\text{s}}_{\text{i}})={\text{μ}}_{\text{i}},{\text{μ}}_{\text{i}}$, in this case, is the SMR log, Var$\left[\text{Y}({\text{s}}_{\text{i}})|\text{Y}(\text{s}{(}_{-\text{i}}))\right]$ is the conditional variance, c_ij_ denote the spatial dependence parameters, in particular ${\text{c}}_{\text{ii}}=0$, c_ij_ is nonzero when s_j_ is close to s_i_. The matrices $\text{C}=({\text{c}}_{\text{ij}})$ and ${\text{Σ}}_{\text{c}}=\text{diag}[{{\text{σ}}_{1}}_{,}^{2}{{\text{σ}}_{2}}_{,}^{2}\cdots ,{\sigma_{\text{n}}}^{2}]$ are obtained and thus ${\text{Σ}}_{\text{Y}}={\left(\text{I}\;\text{C}\right)}^{-1}{\text{Σ}}_{\text{c}}$.

The structure of C is usually specified with a single parameter based on the defined neighborhood matrix, W. C is given by ρ_c_W, where ρ_c_ is the spatial dependence parameter to be estimated. The parameters are estimated by the maximum likelihood method.

Descriptive analyzes were also carried out, through maps of the distribution of SMR by IHD and CBVD, SDI, and NDVI by census tracts. As some census tracts have very small populations, resulting in instability for gross rates, SMR were smoothed by the Bayesian Empirical Global method, whose neighborhood matrix was defined by contiguity.

Statistical analysis and georeferencing were performed in R 3.1.1 and the geoprocessing procedures were performed in the QGIS 2.6.1 program. The study was approved by the Research Ethics Committees of Instituto de Medicina Social of the Universidade do Estado do Rio de Janeiro and the Municipal Health Department of Rio de Janeiro (Opinion 531635).

## RESULTS

A total of 25,959 deaths from IHD or CBVD were reported among people aged over 30 years old, between 2010 and 2012, living in the city of Rio de Janeiro. The 23,800 deaths were georeferenced, resulting in 9.2% of losses. Deaths located in normal tracts, included in the study, totaled 21,689, 11,817 for IHD and 9,872 for CBVD.

Table 1 presents the summary of the descriptive statistics of the variables included in the study. The distribution maps of SMR by IHD and CBVD, SDI and mean NDVI by census tracts in the city of Rio de Janeiro, are presented in Figure (A-D).

**Figure:**
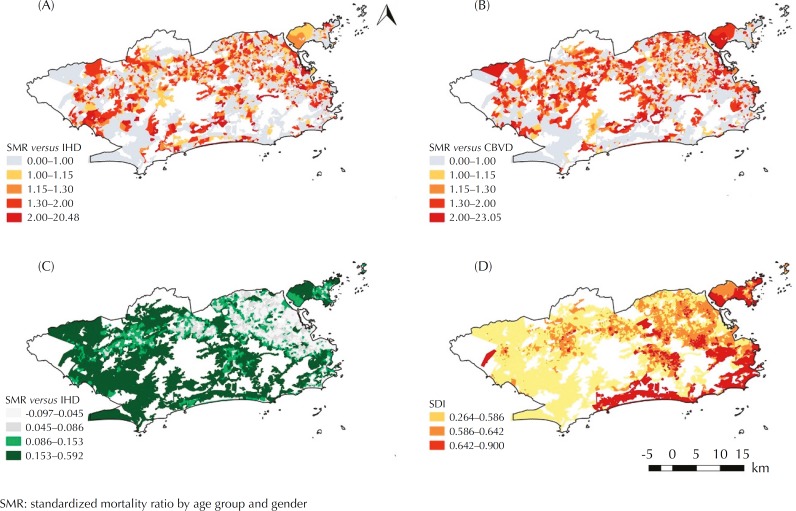
(A) Spatial distribution of the ischemic heart disease (IHD) mortality ratio by normal census tracts in the city of Rio de Janeiro from 2010 to 2012; (B) Spatial distribution of mortality ratio due to cerebrovascular diseases (CBVD), by normal census tracts, in the city of Rio de Janeiro, from 2010 to 2012; (C) Spatial distribution of the Social Development Index (SDI), by normal census tracts, in the city of Rio de Janeiro in 2010; (D) Spatial distribution of the Vegetation Index by Normalized Difference (NDVI), by census tracts, in the city of Rio de Janeiro, from 2010 to 2012.

**Table 1:** Descriptive statistics of the variables included in the analyzes, referring to the normal census tracts of the city of Rio de Janeiro.

Variable		Minimum	1st quartile	Medium	Average	3rd quartile	Maximum
SMR for ischemic heart disease		0.000	0.000	0.707	0.963	1.400	23.030
SMR for cerebrovascular diseases		0.000	0.000	0.648	0.978	1.393	29.950
Social Development Index (SDI)		0.265	0.575	0.608	0.623	0.675	0.900
Density of light roads (m^-1^)		0.0000	0.0100	0.0136	0.0139	0.0174	0.0472
Density of heavy roads (m^-1^)		0.0000	0.0000	0.0000	0.0023	0.0038	0.0276
Normalized Difference Vegetation Index Average							
	All normal tracts	-0.097	0.045	0.086	0.111	0.153	0.593
	Tracts with low SDI	-0.044	0.061	0.061	0.140	0.192	0.576
	Tracts with increased SDI	-0.061	0.039	0.070	0.087	0.120	0.580
	Tracts with high SDI	-0.097	0.041	0.085	0.105	0.154	0.593

SMR: standardized mortality ratio by age group and gender

Spatial distributions of SMR by IHD (Figure, A) and CBVD (Figure, B) did not exhibit any definite pattern. The distribution of SDI (Figure, C) showed a higher concentration of low values between the north and west zones of the city. High values were concentrated in the southern region. The mean NDVI map (Figure, D) showed the concentration of low values in the northern part of the city. The greenest sectors were concentrated between the south and west zones; the latter is the region with the highest number of less urbanized areas, such as granges and farms.

The multivariate analysis (Table 2), adjusted by the SDI, light and heavy road densities and by the indicator of coastal sectors, showed the protective effect of green spaces on IHD mortality in tracts with NDVI above the median. For CBVD mortality, the protective effect was observed only in the tracts with NDVI above the third quartile. Among these greener sectors, with NDVI above the third quartile, the reduction in IHD mortality was 6.7% (95%CI 3.5–9.8) and CBVD was 4.7% (95%CI 1.2–8.0). The SMR were also inversely associated with SDI and directly associated with exposure to air pollution. The autocorrelations of the models, given by the coefficient ρ, were positive, although they were close to zero: 0.085 (0.051; 0.119) for the IHD model and 0.078 (0.004; 0.151) for the CBVD model.

**Table 2:** Coefficients (β) of the autoregressive conditional models for the association between the SMR log, per IHD and CBVD, and the mean NDVI, in the municipality of Rio de Janeiro, 2010 to 2012.

Variable		IHD		CBVD	
		β	95%CI	β	95%CI
Social Development Index		-0.705	-0.812– -0.599	-1.413	-1.569– -1.257
Indicator of coastal tracts		0.014	-0.135–0.164	0.017	-0.084–0.119
Density of light roads		1.958	1.861–2.056	3.328	0.993–5.663
Density of heavy roads		4.606	2.369–6.843	4.798	0.753–8.843
Average NDVI quarters					
	Q2	0.023	-0.008–0.054	0.010	-0.022–0.043
	Q3	-0.043	-0.075– -0.012	-0.011	-0.044–0.022
	Q4	-0.069	-0.101– -0.038	-0.048	-0.083– -0.012

SMR: standardized mortality ratio by age group and gender; IHD: ischemic heart diseases; CBVD: cerebrovascular diseases; NDVI: Normalized Difference Vegetation Index

Regarding the models stratified by tertiles of the SDI (Table 3), the protective effect of green spaces on IHD mortality among the greener sectors (NDVI above the third quartile) in all strata was observed. This protective effect was higher for the sectors with the low SDI. Among the greener sectors, the reduction in mortality was 8.6% (95%CI 1.8–15.0) in the stratum with low SDI, 6.3% (95%CI 1.1–11.2) in the intermediate stratum and 6.7% (95%CI 2.0–11.2) at the highest. For CBVD mortality, the protective effect of exposure was verified only for the greenest sectors of the lowest stratum of the SDI. In these areas, the reduction in mortality was 9.6% (95%CI 2.3–16.5). Significant spatial autocorrelation coefficients among the models with IHD mortality were 0.093 (0.002; 0.184) for the low SDI tracts; and -0.073 (-0.143; -0.002) for tracts with high IDS. Among the models with the CBVD mortality, only the tracts with high SDI had significant autocorrelation and equal to 0.171 (0.104; 0.239).

**Table 3:** Coefficients (β) of the conditional autoregressive models for the associations between the SMR log, per IHD and CBVD, and the mean NDVI, in the city of Rio de Janeiro, 2010 to 2012, stratified by SDI tertiles.

Average NDVI quarters		1st stratum of SDI^a^	2nd stratum of SDI^b^	3rd stratum of SDI^c^
		β (95%CI)	β (95%CI)	β (95%CI)
IHD	Q2	-0.043 (-0.103–0.017)	0.026 (-0.025–0.077)	0.045 (-0.003–0.093)
	Q3	-0.044 (-0.105–0.018)	-0.034 (-0.085–0.018)	-0.040 (-0.089–0.009)
	Q4	-0.090 (-0.163– -0.018)	-0.065 (-0.119– -0.011)	-0.069 (-0.119– -0.020)
CBVD	Q2	0.003 (-0.062–0.068)	0.010 (-0.045–0.065)	-0.001 (-0.046–0.045)
	Q3	-0.011 (-0.078–0.055)	0.005 (-0.050–0.060)	-0.031 (-0.078–0.015)
	Q4	-0.101 (-0.180– -0.023)	-0.020 (-0.077–0.038)	-0.036 (-0.083–0.012)

SMR: standardized mortality ratio by age group and gender; IHD: ischemic heart diseases; CBVD: cerebrovascular diseases; SDI: Social Development Index; NDVI: Normalized Difference Vegetation Index

a Adjusted by the density of light and heavy traffic roads.

b Adjusted by the indicator of coastal tracts, by the density of light and heavy traffic roads.

## DISCUSSION

The results of this study evidenced the protective effect of green spaces on mortality due to IHD and CBVD in the city of Rio de Janeiro, besides the protective effect of the socioeconomic level and the deleterious effect of air pollution. Mortality risks were lower among the greener sectors, with mean NDVI ranging from 0.11 to 0.59.

The city of Rio de Janeiro has greenery around 72% of the households, occupying the 12th position among the Brazilian capitals (Campo Grande is the first with 96.5%, São Paulo is the 10th with 75%, Manaus, Belém and Rio Branco occupy the 25th, 26th, and 27th positions, with 24%, 22.3% and 14% respectively)^d^. The distribution of NDVI in the city of Rio de Janeiro is very uneven, with the northern zone having the lowest rates. However, the city has a recent master plan for afforestation, which is intended to guide actions to expand afforestation^e^, and initiatives such as the Urban Property and Land Tax (IPTU) and municipal seedling nurseries are in the process of being implemented.

The results are consistent with other studies. In an ecological investigation, in two cities in the USA, the lowest risk of stroke mortality was observed in areas with lower pollution, higher income and higher NDVI^14^. In a cross-sectional study conducted in New Zealand, the risk of CVD was lower among residents of regions with a higher proportion of green spaces^15^. This association was maintained even after adjusting for the level of physical activity. A longitudinal study, conducted in Ontario, Canada, found that residents of sites with higher NDVI had lower rates of CVD mortality, among other outcomes^16^. The relationship remained significant even after adjustment for atmospheric pollution. A study carried out in São Paulo observed the inverse association between the total green area per square meter and the risk of CVD among the elderly, regardless of socioeconomic factors and the average income of the dwelling region^17^.

The relationship between green spaces and cardiovascular health can be mediated by the practice of physical activities, by improving psychosocial conditions, such as reducing stress and increasing contact and social cohesion, as well as improving air quality and thermal comfort^6^ ^,^ ^7^.

The practice of physical activities is supposed to be one of the main ways in which the benefits of green spaces manifest in health. The availability of spaces such as parks, gardens and tree-lined streets has the potential to promote physical activity, which is associated with the reduction of numerous precursors of CVD^18^ ^,^ ^19^. The benefits of psychosocial conditions come from contact with the natural space. This impact has positive effects on mental health, such as levels of stress and anxiety, feelings of well-being and increased social interaction, through involvement in physical and social activities^20^ ^,^ ^21^. Psychosocial conditions, in turn, can affect cardiovascular health through pathophysiological and behavioral mechanisms^5^.

Inverse relationships between IHD and CBVD mortality and socioeconomic status, as measured by SDI, were also observed. Cardiovascular morbidity and mortality have been associated with both individual and contextual socioeconomic status^22^. In the analysis stratified by tertiles of SDI, the protective effect of green spaces was higher for the lower socioeconomic level in the case of IHD mortality and observed only for the same stratum in the case of CBVD mortality. These results may be indicating the importance of green exposure among the socioeconomically disadvantaged population. According to Mitchell and Popham^23^ exposure has the potential to reduce health inequities, since the difference in mortality between extreme socioeconomic levels was lower in greener places.

The protective effect of green spaces can also be achieved by reducing air pollution and temperature^6^. However, as air pollution has been adjusted in this study, it is likely that the effects of exposure would be through other pathways. In any case, the associations of atmospheric pollution indicators with IHD and CBVD mortality were positive, in accordance with the results of other studies^24^.

One of the limitations of this study is the exclusion of the census tracts classified as subnormal agglomerates, referring to areas of irregular settlements, where 22% of the population lives. The option was due to the greater occurrence of losses during geo-referencing, mainly due to the urban infrastructure of these localities. Such losses lead to underestimation of mortality rates in these areas. Even in the analysis considering all tracts, not shown in the result, the reduction of the risk of mortality due to IHD and CVD was observed among the greenest census tracts, although it was lower.

Among other limitations, there is the unavailability of information on the prevalence of CVD risk factors at the census tract level, such as smoking, eating habits, hypertension, diabetes, and dyslipidemia. However, according to Diez-Roux^25^, the prevalence of these factors has been related to socioeconomic characteristics of the dwelling place, measured from census information. In this sense, the socioeconomic condition, represented in the present study by the SDI, could be acting as a marker of these determinants.

The measure of exposure, given by the average NDVI, refers to the period in which deaths occurred, disregarding variations throughout life or day, due to migrations and differences in exposure between living and working places. Other aspects related to green spaces that may affect its use were also not considered, such as accessibility, activity opportunities, quality of physical attributes, aesthetic aspects, and safety^7^ ^,^ ^18^.

Despite the limitations, the present study corroborates the importance of contextual factors for cardiovascular health. Especially the protective effect of green spaces on mortality due to IHD and CBVD, mainly in the most deprived areas. So far, few such investigations have been conducted in middle- and low-income countries, especially in Latin America. Future studies with more refined designs that advance the investigation of the mechanisms and mediators of the contextual factors effects are fundamental for a better understanding of these relationships, as well as the effect of different types and characteristics of green spaces. This information is of great relevance in the orientation of health promotion measures in urban space, aimed at controlling the various chronic diseases, focused on both individual and contextual aspects.

## Footnotes

a Ministério da Saúde (BR), Departamento de Informática do SUS - DATASUS. Informações de Saúde (TABNET). Brasília (DF); c2008 [cited 2015 Jan 12]. Available from: http://www2.datasus.gov.br/DATASUS/index.php

b Programa das Nações Unidas para o Desenvolvimento. Atlas do Desenvolvimento Humano no Brasil. Brasília (DF); PNUD; c2014 [cited 2017 Apr 4]. Available from: http://www.atlasbrasil.org.br/2013/pt/

c Weier J, Herring D. Measuring Vegetation (NDVI & EVI). Greenbelt (MD): NASA Earth Observatory; 2000 [cited 2016 Jun 30]. Available from: https://earthobservatory.nasa.gov/Features/MeasuringVegetation/printall.php

d Instituto Brasileiro de Geografia e Estatística. Censo Demográfico 2010: características urbanísticas do entorno dos domicílios. Rio de Janeiro: IBGE; 2010 [cited 2017 Aug 31]. Available from: http://www.ibge.gov.br/home/estatistica/populacao/censo2010/entorno/default_entorno.shtm

e Prefeitura do Rio de Janeiro, Fundação Parques e Jardins. Plano diretor de Arborização Urbana da Cidade do Rio de Janeiro. Rio de Janeiro: FPJ; 2015 [cited 2017 Aug 31]. Available from: http://www.rio.rj.gov.br/web/fpj/plano-diretor-de-arborizacao-urbana
